# Magnetic Shaftless Propeller Millirobot with Multimodal Motion for Small-Scale Fluidic Manipulation

**DOI:** 10.34133/cbsystems.0235

**Published:** 2025-03-12

**Authors:** Yaozhen Hou, Shihao Zhong, Zhiqiang Zheng, Jiabao Du, Ruhao Nie, Qing Shi, Qiang Huang, Huaping Wang

**Affiliations:** ^1^ Intelligent Robotics Institute, School of Mechatronical Engineering, Beijing Institute of Technology, Beijing 100081, China.; ^2^ School of Medical Engineering, Beijing Institute of Technology, Zhuhai 519088, China.; ^3^ Department of Biomedical Engineering, City University of Hong Kong, Hong Kong 999077, China.; ^4^ Key Laboratory of Biomimetic Robots and Systems (Beijing Institute of Technology), Ministry of Education, Beijing 100081, China.

## Abstract

Magnetic miniature robots have shown great potential in biomedical applications in recent years. However, a challenge remains in which it is difficult for magnetic miniature robots to achieve balanced capabilities for multimodal locomotion and fluidic manipulation in various environments. Here, we report a magnetic shaftless propeller-like millirobot (MSPM) that possesses the capabilities of rotating-based multimodal 3-dimensional motion and cargo transportation with untethered manipulation. The MSPM utilizes the propulsion and pumping capabilities of the propeller structure to achieve fluidic manipulation. The shaftless propeller structures are designed to achieve omnidirectional locomotion through rolling, propelling, and tumbling. Additionally, the shaftless 3-blade propeller is used to perform a pumping function to achieve controllable transportation of fluids and particles. We anticipate that the MSPM holds great potential as a minimally invasive device for thrombosis treatment and targeted medicine delivery.

## Introduction

Magnetic miniature robots [1–5] have been receiving increasing attention for many applications, such as micromanipulation [6], transportation [7], environmental remediation [8], and biomedical applications [9]. They are capable of remote locomotion, shape morphing, and fine operation in confined environments under the actuation of multiple stimuli (e.g., pH, temperature, light, and electric and magnetic fields) [10–17]. Among them, magnetic actuation has been widely employed owing to its untethered actuation, real-time response, and uniform control [18]. For instance, helical robots have demonstrated the ability to achieve 3-dimensional (3D) motion in viscous liquids [19], strip-like robots can perform multimode motion in confined environments [20], and round-like robots are capable of transportation in narrow environments [21]. While these robots exhibit strong advantages in certain environments or task scenes, achieving balanced capabilities of locomotion or manipulation in on-ground environments, in-water environments, and other typical biomedical environments remains a challenge for magnetic miniature robots.

Rotating-based motions, including rolling [22], propelling [23], and tumbling [24], are widely employed in robotic design to achieve in-water and on-ground locomotion. Compared with other motion mechanisms, rotating-based motions, such as corkscrew motion, wobbling, and crawling, which are commonly used in miniature robotics, have higher movement efficiency [25–27]. The highly symmetric configuration of these robots not only enables steady rolling motion and flexible steering motion but also enables effective underwater propulsion. For biomedical applications, the development of an untethered magnetic-field-actuated miniature robot capable of integrating rotation-based locomotion and manipulation is important and beneficial, particularly in the treatment of diseases affecting the blood system and urinary system. While rotary motions are effective for generating locomotion in both in-water and on-ground environments, a skillfully designed structural configuration of the robot for a distinct motion mechanism is essential for achieving precise omnidirectional propulsion and efficient transportation of fluids and particles.

An engineered thruster or propeller, comprising a central hub and radially arranged blades, converts rotational motion into thrust when operating in air or water, thereby facilitating effective forward propulsion and agile steering [28–30]. However, the traditional propeller relies on actuation of the central hub’s rotation, which requires an external motor for the power supply and supporting structures to minimize vibration. These factors limit the structural design and fabrication of miniature robots for biomedical applications. Existing fabrication methods, including ultraviolet lithography, laser cutting, and direct laser writing (DLW), are widely utilized to fabricate magnetic miniature devices with simple geometries and limited magnetization [31–34]. However, a robot that is composed of complex and unsupported 3D geometry, multiple materials, and enough magnitude of magnetization is challenging to fabricate via one technology. Therefore, there is a pressing need to explore fabrication methodologies that offer fast-prototyping, low time–cost, and batching fabrication capabilities.

In this work, we report a rotating-based magnetic miniature robot capable of multimodal locomotion and liquid/particle transportation under magnetic field actuation. The fabrication combines 2-photon polymerization (2PP) DLW and molding to fabricate an actuation part and a supporting part with magnetic and nonmagnetic elastomeric composites. The robot integrates different parts of one body, enabling multimodal locomotion and fluid manipulation in different environments. The robot can move on unstructured ground through rolling and tumbling and swim in liquids through rotating-based propulsion. Furthermore, the rotating-based propulsion provides a pumping mechanism for liquid and particle transportation. The integration of multimodal motion and fluidic micromanipulation enables the use of a rotating-based magnetic miniature robot as a promising tool in minimally invasive thrombosis treatments and targeted medicine delivery.

## Materials and Methods

### Fabrication of the magnetic shaftless propeller-like millirobot

**Fig. 1.**
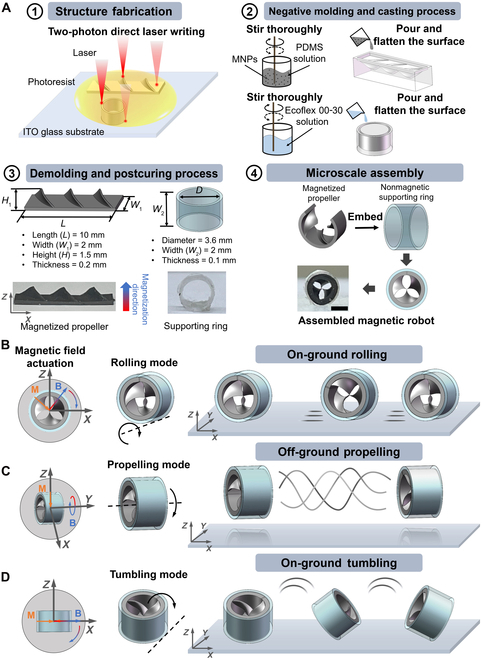
Fabrication and motion mechanism of the magnetically actuated shaftless propeller-like millirobot. (A) Fabrication process of the shaftless propeller-like millirobot, including positive structures’ fabrication by 2-photon polymerization (2PP), negative mold fabrication and casting process of the robot, demolding of different parts and magnetization of the magnetic part, and assembly of the individual parts of the robot. Scale bar: 2 mm. (B) Rolling mode actuated by a rotating field B. (C) Propelling mode in water. (D) Tumbling mode on the ground. ITO, indium tin oxide; PDMS, polydimethylsiloxane.

The robot is inspired by the architecture of an engineered shaftless water pump propeller. The magnetic part of the magnetic shaftless propeller-like millirobot (MSPM) was made from polydimethylsiloxane (PDMS; Sylgard 184, Dow Inc.) embedded with NdFeB microparticles. The nonmagnetic ring-shaped supporting part was made from silicone rubber of Ecoflex 00-30. The final structure was made by embedding magnetic propeller into the ring-shaped supporting structure via tweezers, as shown in Fig. 1A.

### Fabrication of the ferromagnetic propeller-like part

Prior to the fabrication of the magnetic part, a propeller mold was printed using Photonic Professional GT2 (Nanoscribe GmbH) with commercial resin IP-S. The mold featured 3 propellers, each measuring 1.3 mm in height and 2 mm in width, with a tilt angle of 45° and backing layer dimensions of 10 mm × 2 mm × 0.2 mm. After the printing, the master mold was rinsed in propylene glycol monomethyl ether acetate (PGMEA; Aladdin Inc.) solution for 20 min, followed by rinsing in isopropyl alcohol for 2 min to remove the uncured solution. Then, the PDMS (Sylgard 184 Silicone Elastomer) solution (mass ratio of the monomer to the curing agent, 10:1) was poured onto the mold and then degassed for 1 h. The mold was then cured at 85 °C on a hot plate for 1 h, allowing the negative mold to be carefully peeled off from the master mold. To fabricate the ferromagnetic propeller, we first mixed the neodymium–iron–boron particles (NdFeB, average diameter 10 μm, Magnequench GmbH) and PDMS in a plastic container. The mass ratio of the monomer to the curing agent in PDMS was 12:1. The final mass ratio of the NdFeB to the PDMS was 1:1. The mixed polymer was poured onto the propeller mold and then degassed for 45 min at 25 °C. Subsequently, the excess PDMS was scraped off with a razor blade and then cured at 85 °C on a hot plate for 1 h. Once the magnetic propeller structure had cooled to room temperature, it was rinsed in an ethanol solution and carefully detached from the mold using tweezers.

### Fabrication of the nonmagnetic ring-shaped supporting part

A supporting part mold was also printed via 2PP, with dimensions of 3.6 mm in diameter, 2 mm in width, and 0.1 mm in thickness. After the printing, the mold was soaked in PGMEA solution for 12 min and then soaked in isopropyl alcohol for 1 min to remove the unpolymerized resin. The mold was then subjected to a cleaning process and surface activation treatment using Ease Release 200 (Smooth-On Inc.) to enhance the mold release properties. Excess release agent was meticulously wiped off with a rag. The PDMS solution (10:1) was poured onto the mold and then degassed for 1 h. The mold was then cured at 85 °C on a hot plate for 1 h, after which the negative mold was slightly peeled off from the master. A light coating of the release agent was sprayed onto the ring-shaped negative mold, and the thoroughly mixed soft polymer of Ecoflex 00-30 (Smooth-On Inc.) was poured into the ring-shaped mold and then degassed for 15 min at 4 °C. Subsequently, the excess Ecoflex 00-30 solution was scraped off using a razor blade and then cured at 65 °C on a hot plate for 3 h. After the nonmagnetic ring-shaped supporting structure was cooled to room temperature, it was rinsed in an ethanol solution and peeled off from the molds separately with tweezers.

### Magnetic actuation system

The customized electromagnetic coil actuation system is composed of 3 pairs of orthogonally configured Helmholtz coils that can generate uniform magnetic fields (30 mT) within a 3D space of 25 mm in diameter. The setup is controlled via an STM32 card connected to a personal computer. Eight motor drivers (ESCON 50/5 MAXON) are powered by a power supply (LRS-450-48 MEAN WELL) and are used to actuate the coils. Two cameras (DVC830, OVT) are used to achieve simultaneous visualization and recording. The sample can achieve multimodal motion in the workspace under rotating magnetic field actuation. The motion velocities of the robot are determined by the magnitude of the magnetic field strength and frequency, and its moving direction is determined by the magnetic field direction. The magnetic actuation experiments were conducted in opaque plastic plates, a 3D-printed maze model, and a cylindrical acrylic box.

### Computational fluid dynamics simulations

Qualitative analysis of the differences induced by the robot’s propeller was conducted through computational fluid dynamics (CFD) simulations using the Ansys Fluent software (ANSYS, Inc., USA).

## Results

### Magnetically actuated shaftless propeller-like millirobot (MSPM)

We present a miniature magnetic pumping robot inspired by the design of an engineered shaftless water pump propeller [35]. It is fabricated by using the 2PP DLW and molding methods. The MSPM is composed of a ferromagnetic rollable propeller (length, *L* = 10 mm; width, *W* = 2 mm; height, *H* = 1.5 mm; thickness, *T* = 200 μm; and tilt angle, θ = 45°) as the main actuation component and a nonmagnetic ring-shaped cylinder (*D* = 3.6 mm in diameter, *W* = 2 mm in width, and *T* = 100 μm in thickness,) as the supporting component. The combined structure enables multimodal locomotion and in-water propulsion. Fig. 1A shows the fabrication process of the different functional parts and the assembly process. Prior to assembly, the magnetic propeller was subjected to magnetization along the *z* axis with a custom-designed single-axis electromagnet system, which generated a homogeneous 1-T field.

To achieve locomotion of rolling, propelling, and tumbling, the MSPM relies mainly on actuation of the rotating magnetic field. In this study, the MSPM is subjected mainly to magnetic torques, which can be expressed as follows:

T=VM×B (1)

where *V* represents the volume of the robot, ***M*** is the magnetization (A/m), and ***B*** is the magnetic field flux density (T). When the magnetic field rotates, the magnetization direction of the MSPM always tends to align with the direction of the applied magnetic field ***B***, leading to continuous rotation for rolling, propelling, and tumbling. Fig. 1B to D shows 3 motion modes of the robot. Fig. 1B and D show schematics of the rolling and tumbling motion of the robot on the ground, respectively. They are achieved by adjusting the rotating direction of the magnetic field to make it parallel or perpendicular to the longitudinal axis of the robot. Fig. 1C shows a schematic of the propulsion mode of the robot in liquids. This can be achieved by changing the direction of the rotating magnetic field to make it consistent with the longitudinal axis of the robot.

### Self-adaptive motion in unstructured environments

**Fig. 2.**
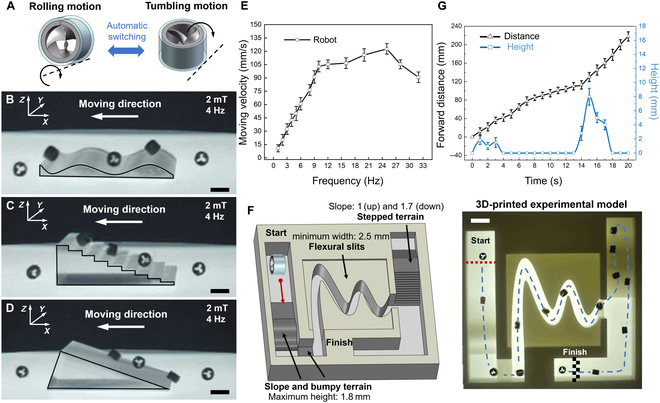
Self-adaptive motion in unstructured environment. (A) Self-adaptive motion mechanism of the robot. Under the actuation with 2 mT and 4 Hz, the robot moves through different terrains by self-adaptive motion, including undulatory terrain (B), stair-like terrain (C), and inclined terrain (D). Scale bar: 5 mm. (E) Characterization of the moving performance of the robot on the ground. Each bar represents the mean value ± standard error of the mean from 10 independent experiments. (F) Self-adaptive motion of the robot in a 3-dimensionally (3D) printed maze model. Scale bar: 5 mm. (G) Characterization of the moving performance in an artificial model. Each bar represents the mean value ± standard error of the mean from 10 independent experiments.

The multimodal motion of the MSPM in unstructured environments is crucial for demonstrating adaptability in different applications [36,37]. Most existing robots can change their morphologies [38,39] or actuation modes [11,40] when encountering different environments, such as undulatory terrains, stair-like terrains, and slopes. Unlike robots, which require specific adjustments to their motion postures, our proposed MSPM can effortlessly perform dexterous rotating-based motions. Once the moving direction of the robot is determined, the rotating magnetic field ensures that the robot moves in the desired direction, as the MSPM’s moving direction is independent of its initial orientation. When the robot faces obstacles or rough surfaces, it can automatically switch motion modes between rolling and tumbling, as shown in Fig. 2A. This ensures that the robot effectively adapts to the terrains while maintaining the moving direction without changing the magnetic field. Fig. 2B shows the robot’s initiation of motion under a rotating magnetic field of 2 mT and 4 Hz, with the initial longitudinal axis of the robot parallel to the ground. As the magnetic field rotates counterclockwise in the *X*–*Z* plane, the robot rolls on the undulatory terrain and is disturbed by the roughness of the surface to automatically switch its motion mode from rolling to tumbling without changing the rotating direction of the field. Fig. 2C illustrates the robot’s capability to pass through stair-like terrain by automatically switching its motion mode between rolling and tumbling. In this process, the robot rotates counterclockwise under the actuation of the magnetic field, effectively overcoming the obstacle. Fig. 2D demonstrates that the robot can pass through inclined terrain through self-adaptive motion (see Movie S1). The characterization of the moving velocity with respect to the magnetic field rotating frequency during ground-based self-adaptive motion is shown in Fig. 2E. The MSPM reaches a maximum moving velocity of 122.7 mm/s under a rotating magnetic field with strength ***B*** = 2 mT and frequency *f* = 25 Hz.

To further test the self-adaptive motion capabilities, the robot was actuated in a 3D-printed maze that featured inclined and bumpy terrain, with the maximum height reaching 1.8 mm (half the height of the robot), flexural slits with a minimum width of 2.5 mm, and stepped terrain, as shown in Fig. 2F (see Movie S2). The experiment demonstrates that the robot is capable of successfully moving along a predetermined path through complicated, diverse, and unstructured terrain by leveraging its effective self-adaptive motion (rolling and tumbling). Fig. 2G shows the variation in moving distance and height as a function of time (s) under magnetic actuation at field strength of B=2mT and f=4Hz. These experiments demonstrate the robustness of the robot’s self-adaptive motion capabilities. The self-adaptive motion of the robot in different terrains can markedly reduce the control complexity, which is particularly beneficial for an actuating robot to move on complex unpredictable environments.

### Magnetic actuation and predetermined path following motion

**Fig. 3.**
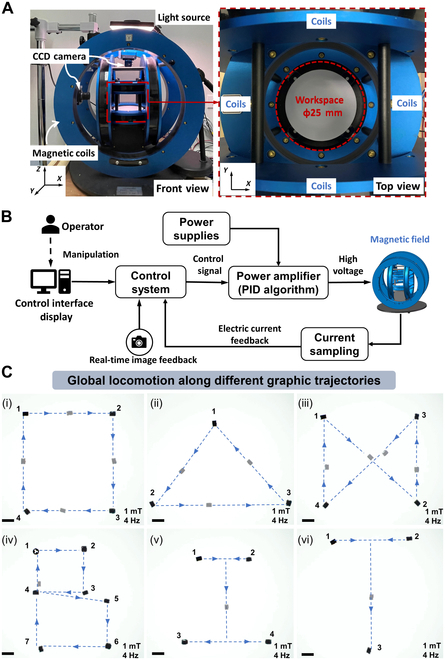
Magnetic actuation and predetermined path following motion. (A) Schematic of the customized 3D Helmholtz electromagnetic coil actuation system. (B) Control diagram of the magnetic actuation for the shaftless propeller-like millirobot. (C) Predetermined path following of the robot, including a square shape, a triangle shape, crosslines, and the letters “BIT”. Scale bar: 5 mm. CCD, charge-coupled device; PID, proportional–integral–derivative.

Fig. 3A shows the customized 3D Helmholtz electromagnetic coil actuation system that can generate multiple types of magnetic fields, such as rotating magnetic fields, oscillating magnetic fields, and gradient magnetic fields. In this work, the robot is mainly subjected to a magnetic torque ***T*** generated by a rotating magnetic field. At any point *P* in the working space, the magnetic field can be represented by a vector $B_{e}\left(P\right)$, and its magnitude varies linearly with the current passing through the coil, so it can be described as

$$B_{e}\left(P\right)={\tilde{B}}_{e}\left(P\right)i_{e}$$ (2)

The subscript *e* denotes the contribution due to activation of the *e*th solenoid. For Helmholtz coils, the contributions of the individual fields are decoupled and the fields can be precomputed individually and then superimposed linearly. Therefore, the magnetic field at a point in the workspace can be expressed as

$$B\left(P\right)=\sum_{e=1}^{n}B_{e}\left(P\right)=\sum_{e=1}^{n}{\tilde{B}}_{e}\left(P\right)i_{e}$$ (3)

$$B\left(P\right)=\left[{\tilde{B}}_{1}\left(P\right)\;\cdots \;{\tilde{B}}_{n}\left(P\right)\right]\left[\begin{array}{c} i_{1}\\ \vdots \\ i_{n} \end{array}\right]=\beta \left(P\right)I$$ (4)

The torque can be described as

$$T=\left[\begin{array}{c} \mathrm{Sk}\left(M\right)\beta \left(P\right)\\ \begin{array}{c} M^{T}\beta_{x}\left(P\right)\\ \begin{array}{c} M^{T}\beta_{y}\left(P\right)\\ M^{T}\beta_{z}\left(P\right) \end{array} \end{array} \end{array}\right]\left[\begin{array}{c} i_{1}\\ \vdots \\ i_{n} \end{array}\right]=A_{T,F}\left(M,P\right)I$$ (5)

For each robot pose, *n* coil currents are mapped to torque through a 6 × *n* matrix $A_{T,F}\left(M,P\right)$. The desired torque can be obtained by a pseudo-inverse solution:

$$I=A_{T,F}{\left(M,P\right)}^{+}T_{\mathrm{des}}$$ (6)

Taking the generation of a rotating magnetic field on the *xoz* plane as an example, the magnetic field rotating around the *y* axis as $B^{y}$,

By=BxByBz=Bmsin2πft0Bmcos2πft (7)

where $B_{m}$ is the peak value of the oscillating magnetic field and where *f* is the rotating frequency. The magnetic field to the input current of the coil is

Iy=IxIyIz=Im1sin2πft0Im2cos2πft (8)

Fig. 3B shows the control block diagram of a feedback control system. A proportional–integral–derivative control method is used to transmit the control signal from the upper computer to the lower computer and generate the desired current signal to adjust the magnetic field output. The magnetic field accuracy is indirectly controlled by current feedback control.

Fig. 3C (i) to (vi) shows that the robot is actuated to move along predetermined paths, which include a square shape, a triangle shape, crosslines, and the letters “BIT”. All of the tests demonstrate the robot has dexterous and robust moving performance along complicated paths, which is essential for future use to perform precise manipulation or targeted delivery (see Movie S3).

### CFD simulation of the MSPM and ring-shaped robot

**Fig. 4.**
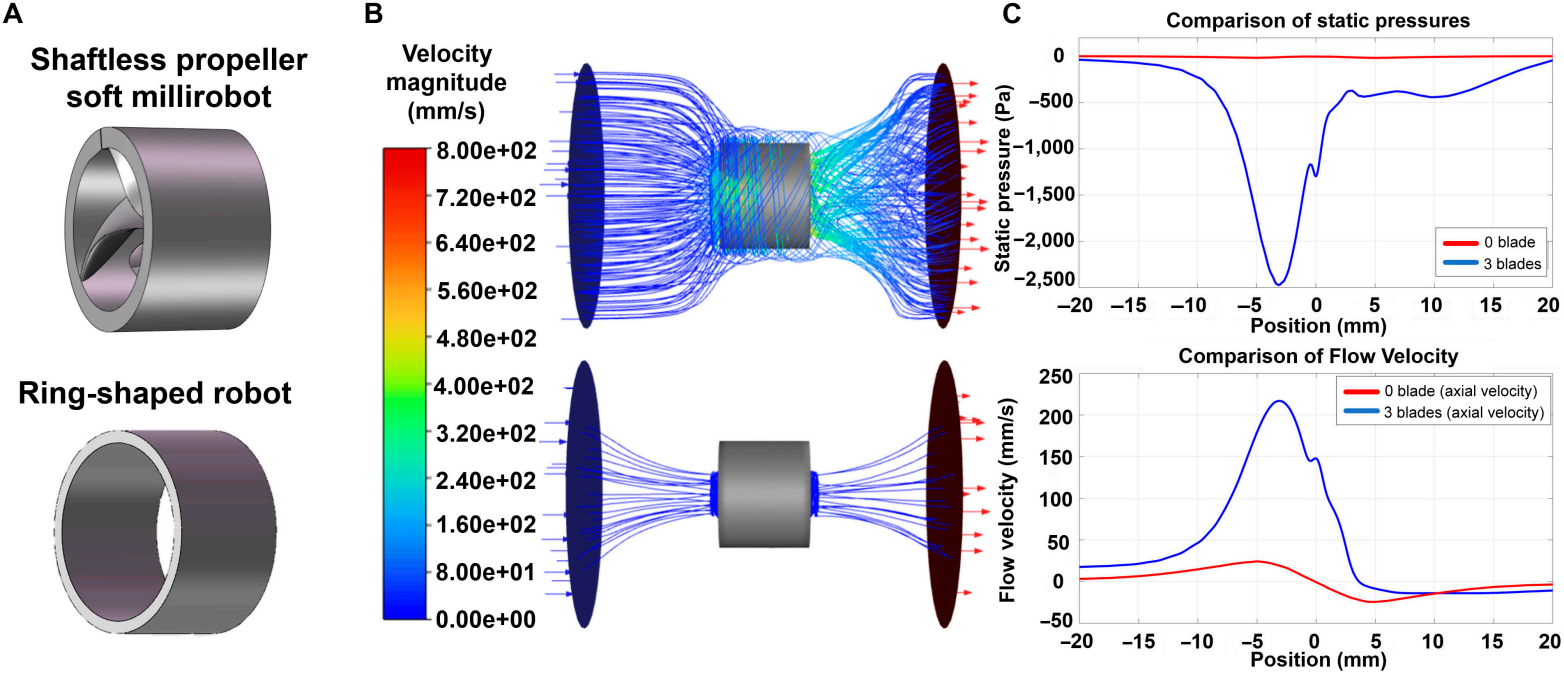
Comparison of the streamlines, normalized pressures, and normalized velocities of the magnetic shaftless propeller-like millirobot (MSPM) robot and the ring-shaped robot in computational fluid dynamics (CFD). (A) Schematic of the shaftless propeller-like millirobot (up) and the ring-shaped robot (down). (B) CFD simulation streamlines of the shaftless propeller-like millirobot (up) and the ring-shaped robot (down). (C) Comparison of normalized velocities and normalized pressures for the shaftless propeller-like millirobot (up) and the ring-shaped robot (down).

A CFD simulation was conducted to qualitatively investigate the propulsion mechanism of the internal propeller within the MSPM. Fig. 4A shows the 3D model of the MSPM and the ring-shaped robot. The external fluid flow is set as a static field. As shown in Fig. 4B, for the robot with an internal propeller, the fluid streamlines encircle the body and pass through the rotating axis of the body. The internal propellers facilitate the generation of a centralized backward propulsion flow that is expelled through the hole. In contrast, the ring-shaped robot generates no fluid flow through its body. A comparative analysis of the gauge pressure between the MSPM robot and the ring-shaped robot indicates a prominent decrease along the MSPM’s rotating axis within the body, as shown in Fig. 4B. The pressure drop proves a decrease in swimming resistance, thereby increasing propulsion velocity. Moreover, the flow velocity in the simulation results demonstrates that the propellers can generate fluid flow at higher speeds, which in turn can produce a higher backward propulsion force to drive the robot to move forward, as shown in Fig. 4C.

### Spinning-based swimming under water and fluid transportation

Screw propellers can transmit power by converting rotational motion into thrust, which has high propulsion efficiency, stable transmission, and robust governing [41,42]. Thus, propeller systems are widely used as propulsion units in ship machinery and underwater robots for pipeline inspection, safe rescue, and resource exploration. Traditional propellers typically consist of a central hub and radially distributed blades, necessitating complex shell and supporting structure for integration. In contrast, the shaftless propeller system employs a direct propulsion mechanism, which is characterized by low resistance and high propulsion efficiency.

**Fig. 5.**
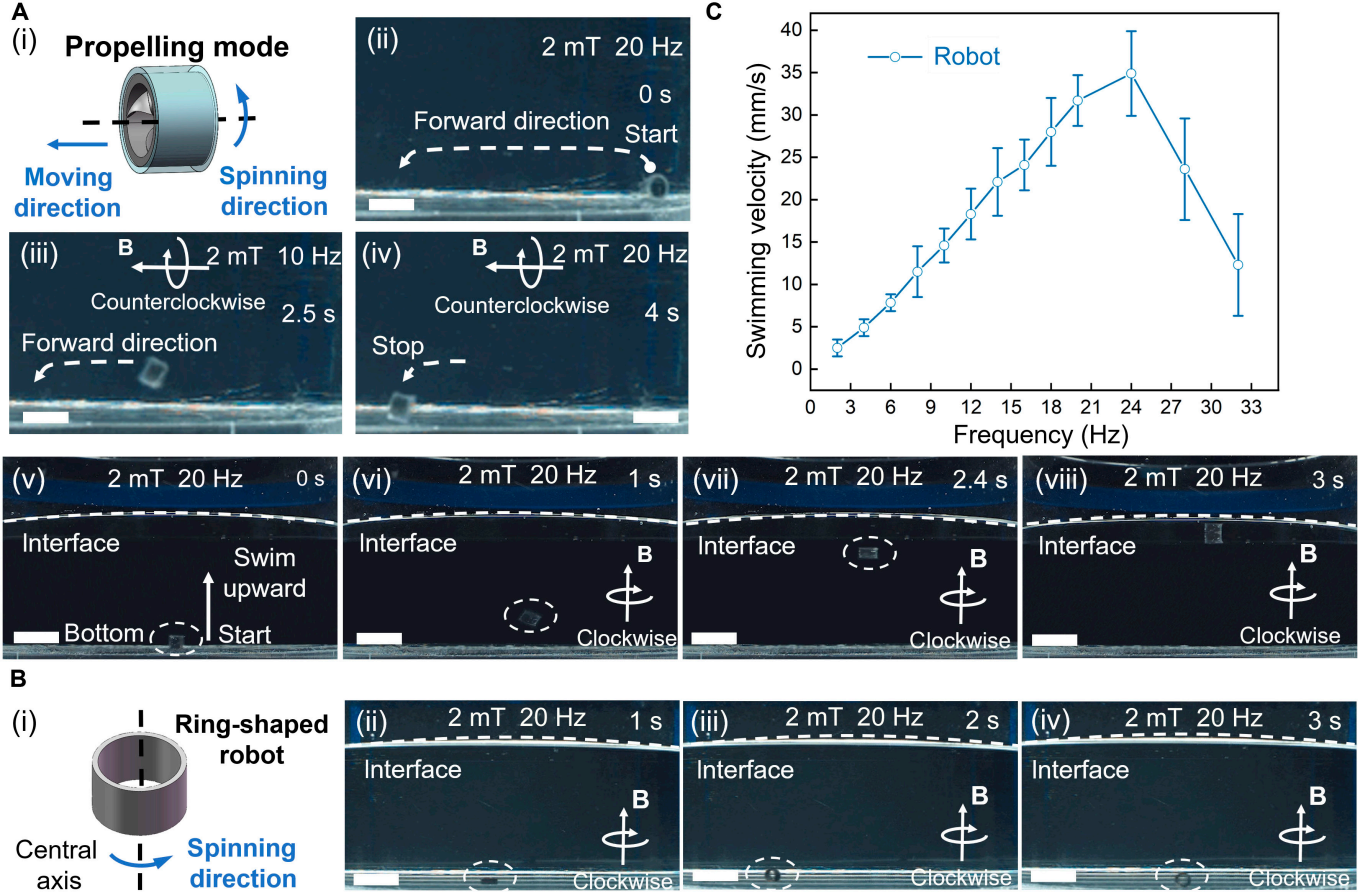
Spinning-based swimming under water. (A) Spinning-based swimming motion of the MSPM. (i) Spinning-based swimming mechanism of the MSPM. (ii) The robot starts to swim under the rotating magnetic field actuation with 2 mT and 10 Hz. (iii) The robot gets off the ground by propulsion and swims from the right side to the left side. (iv) The robot reaches the target point. (v) The robot starts to swim along the vertical axis in a water jar. (vi) The robot swims upward at a magnetic field of 2 mT and 20 Hz. (vii) The robot continues to swim upward in water. (vii) The robot finally reaches the air–water interface under the magnetic field actuation. Scale bar: 5 mm. (B) Spinning-based swimming motion of the ring-shaped robot. (i) Spinning-based swimming mechanism of the ring-shaped robot. (ii) The ring-shaped robot starts to swim under the rotating magnetic field actuation with 2 mT and 20 Hz. (iii) Under the magnetic field actuation, the ring-shaped robot spins in situ. (iv) The ring-shaped robot still spins in situ and never leaves the bottom. (C) Characterization of the swimming performance of the MSPM in the water. Each bar represents the mean value ± standard error of the mean from 10 independent experiments.

For our magnetically actuated shaftless propeller-like millirobot, the propeller blades can be directly arranged on the supporting circular ring, which reduces the difficulty of design and fabrication. We demonstrate that the robot is capable of achieving underwater swimming through spinning-based motion, leveraging the propeller’s structural features to interact effectively with water. When subjected to a counterclockwise rotating magnetic field at a frequency of *f*, the MSPM robot spins and generates a propulsion force to the right by pushing water backward using the blades (Fig. 5A (i)). As shown in Fig. 5A (ii), the MSPM is actuated under a rotating magnetic field of 2 mT and 10 Hz to achieve swimming motion. In this swimming process, the MSPM lifts off the ground and swims from the right side to the left side over a duration of 4 s (Fig. 5A (iii) and (iv)) (see Movie S4). In addition, we also evaluated the MSPM’s swimming performance along the vertical axis in a water jar. Fig. 5A (v) demonstrates the robot’s actuation under a higher magnetic field rotating frequency, initiating its swimming from the bottom of the jar at a 45° right pitching angle to minimize the influence of gravity. The robot can gradually approach the air–liquid surface at a magnetic field rotating frequency of 20 Hz (Fig. 5A (vi) to (viii)) (see Movie S5). For comparison, we evaluated the spinning-based motion of the ring-shaped robot in the same water jar (Fig. 5B (i)). In Fig. 5B (ii) to (iv), when a clockwise rotating magnetic field is applied along the *y* axis, the ring-shaped robot just spins at the bottom instead of lifting and swimming in the water (see Movie S6). Fig. 5C shows the measured swimming velocity of the MSPM. It is observed that the swimming speed of the MSPM increases with the rotating frequency of the magnetic field, which reaches a maximum moving velocity of 34.9 mm/s at *B* = 2 mT and *f* = 24 Hz. The spinning-based swimming experiments demonstrate that the shaftless propeller-like millirobot exhibits effective propelling capabilities.

**Fig. 6.**
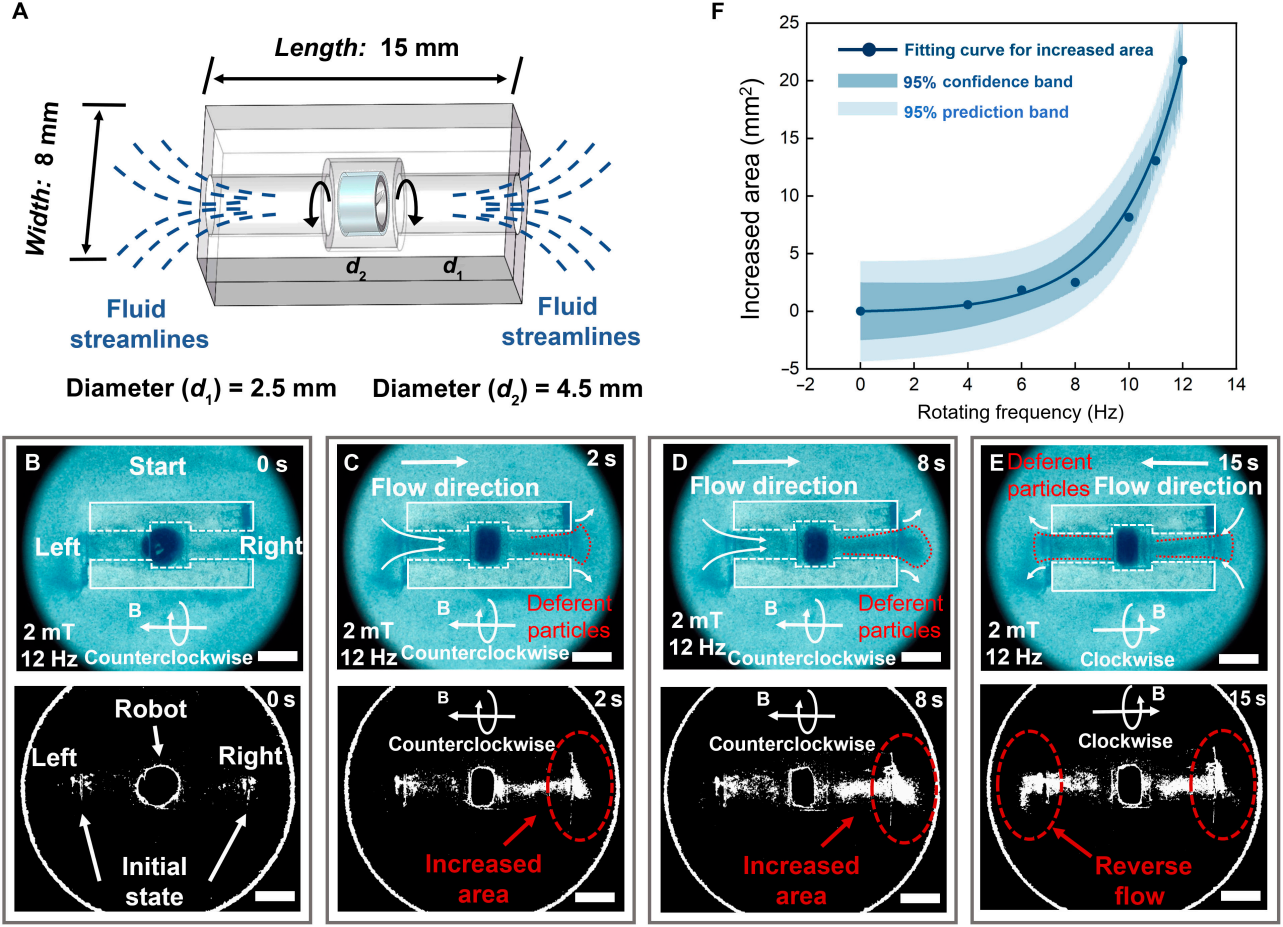
Spinning-based fluid transportation. (A) Schematic of cargo transportation in a narrow environment. (B) The robot is put into a 3D-printed artificial tube to test its capability of cargo transportation (up). Binarization results of the transported fluids at 0 s (bottom). (C) The robot spins at a magnetic field of 2 mT and a frequency of 12 Hz, and the fluids within mica powder begin to flow. Binarization results of the transported fluids at 2 s (bottom). (D) Mass of fluids and mica powder flow through the robot to the right opening of the tube. Binarization results of the transported fluids at 8 s (bottom). (E) The fluids with mica powder flow back to the left opening along the previous trajectory by reversing the magnetic field rotating direction. Binarization results of the transported fluids at 15 s (bottom). Scale bar: 5 mm. (F) Relationship between flow velocity caused by the robot’s rotation and rotating frequency.

The MSPM is capable of transporting fluids and particles within confined environments, leveraging the propulsion capabilities of the internal propeller. We demonstrate that the robot can transport fluid and particles through a 3D-printed artificial tube by spinning-based motion and a propeller-enabled pumping mechanism. The artificial tube, constructed via 3D additive manufacturing with acrylic resin, has dimensions of 15 mm in length, 8 mm in width, 2.5 mm in outer diameter, and 4.5 mm in inner diameter, as shown in Fig. 6A. The inner cylindrical groove provides enough space for the robot’s spinning. When the robot spins, the outer fluids can be sucked into the tube and flow from one side to the other side. Fig. 6B shows the spinning-based motion of the robot in a dish with rheoscopic fluid, including 5 g/ml blue mica powder in deionized water, to visualize the flow. At the beginning, the rheoscopic fluid is evenly filled in the water dish. When the magnetic field rotates counterclockwise at 2 mT and 12 Hz, the robot rotates quickly in the circular groove and generates a fluid stream by the propulsion of the propeller (Fig. 6C). The rheoscopic fluid with mica powder enters into the left opening and gradually flows through the robot’s body along its axis. Fig. 6D shows that the amount of fluid with mica powder is directed toward the right opening of the tube. Then, upon reversing the rotating direction of the magnetic field, the robot rotates in a clockwise direction, resulting in reverse suction for fluid transportation. As shown in Fig. 6E, the fluid with mica powder flows back to the left opening along the previous trajectory. To provide an intuitive display of the transported fluids facilitated by the MSPM, a sequence of the binarization results of the transported fluids is shown at the bottom of Fig. 6B to E. The results indicate an obvious increase in fluids at both ends of the tube over time. Fig. 6F demonstrates that the increased area of the transported fluids is nonlinearly correlated with the rotating frequency of the robot. For a tube in this paper with a cross-sectional area of 1.5625π (mm^2^), the relationship between the flow velocity caused by the robot’s rotating and rotating frequency is $y=0.03\times \left(e^{\left(x/2.32\right)}-1\right)$. The experimental results indicate that the robot equipped with inner propellers exhibits superior fluid dynamic interaction, thereby demonstrating the efficient and stable performance of the MSPM in the transportation of fluid or cargos (see Movie S7).

## Discussion

Here, we demonstrated an untethered robot that employs a shaftless propeller structure and is magnetically actuated to achieve multimodal motions and fluidic manipulation. Leveraging the interaction between the robot’s inner propeller feature and external environments, the robot is capable of performing multiple adaptive motion modes, including rolling, propelling, and tumbling, in different terrains. In addition, the robot demonstrated directed fluidic transportation through a spinning-based pumping mechanism facilitated by its shaftless propeller structure. A nonlinear relationship between the flow velocity induced by the robot’s rotation and the rotating frequency was observed. Experiments conducted in 3D-printed artificial tube highlighted the potential applications of our robot in complex biomedical environments, such as in the treatment of the vascular system and gastrointestinal tract. Furthermore, the proposed concept of a rotating-based shaftless propeller-like millirobot can be scaled up or down for broader applications. The proposed robot allows for the integration of various components within its body, such as cavities and cameras, enabling a range of biomedical applications, including but not limited to microbial sampling and endoscopy. We anticipate that MSPMs could be considered implantable vascular stents to maintain the delivery of nutrients and normal function of cells or organs by adjusting the blood flow and flow velocity. Moreover, the MSPM could also be considered a potential device for diagnosis and treatment in biomedical applications with better functionality and less damage to patients.

## Data Availability

The data are freely available upon request.
