# Supplementary material for: Magnetic Shaftless Propeller Millirobot with Multimodal Motion for Small-Scale Fluidic Manipulation
Source: Cyborg Bionic Syst. 2025 Mar 12;6:0235. doi: 10.34133/cbsystems.0235 (PMC11903028; doi:10.34133/cbsystems.0235)
Supplement: Supplementary 1 — Movies S1 to S7 Fig. S1 [file cbsystems.0235.f1.zip › Supplementary Information.docx]

**Supplementary Information**

**Title**

Magnetic Shaftless Propeller Millirobot with Multimodal Motion for Small-Scale Fluidic Manipulation

**Authors**

Yaozhen Hou^1,2^, Shihao Zhong^2^, Zhiqiang Zheng^3^, Jiabao Du^2^, Ruhao Nie^2^, Qing Shi^1^, Qiang Huang^1^ and Huaping Wang^1*^

**Affiliations**

^1^ Key Laboratory of Biomimetic Robots and Systems (Beijing Institute of Technology), Ministry of Education, Beijing 100081, China.

^2^ Intelligent Robotics Institute, School of Mechatronical Engineering, Beijing Institute of Technology, Beijing 100081, China.

^3^ Department of Biomedical Engineering, City University of Hong Kong, 999077 Hong Kong, China.

*Address correspondence to: wanghuaping@bit.edu.cn

**Movement and fluid transportation in plasma**


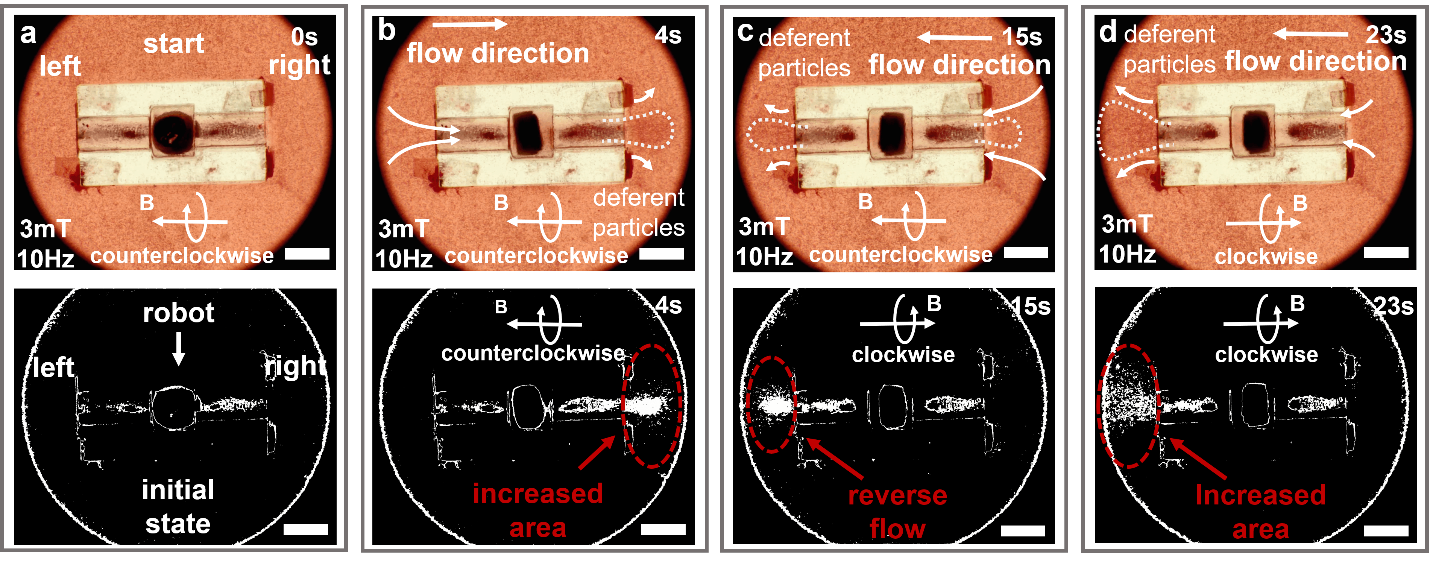
We have demonstrated that the MSPM is capable of transporting fluids and particles in plasma (MP20018, Shanghai yuanye Bio-Technology Co., Ltd), which was made of mixture sheep blood and rheoscopic fluid. Fig. S1(a-d) show the results of the transported fluids and particles facilitated by the MSPM in the miniature artificial tube. And the binarization results of the transported fluids and particles are shown in the bottom of Fig. S1(a-d).

Fig. S1. Spinning-based fluid transportation in plasma of sheep. (a) The robot is put into an 3D-printed artificial tube to test its capability of the cargo transportation (up). Binarization results of the transported fluids at 0s (bottom). (b) The robot spins at a magnetic field of 3 mT and a frequency of 10 Hz, and the fluids within mica powder begin to flow. Binarization results of the transported fluids at 4s (bottom). (c) The fluid with mica powder flow back to the left opening along the previous trajectory by reversing the magnetic field rotating direction. Binarization results of the transported fluids at 15s (bottom). (d) Mass of fluids and mica powder flow through the robot to the left opening of the tube. Binarization results of the transported fluids at 23s (bottom). Scale bar: 5 mm.
