# Supplementary figures and images for: Magnetic Shaftless Propeller Millirobot with Multimodal Motion for Small-Scale Fluidic Manipulation
Source: Cyborg Bionic Syst. 2025 Mar 12;6:0235. doi: 10.34133/cbsystems.0235 (PMC11903028; doi:10.34133/cbsystems.0235)

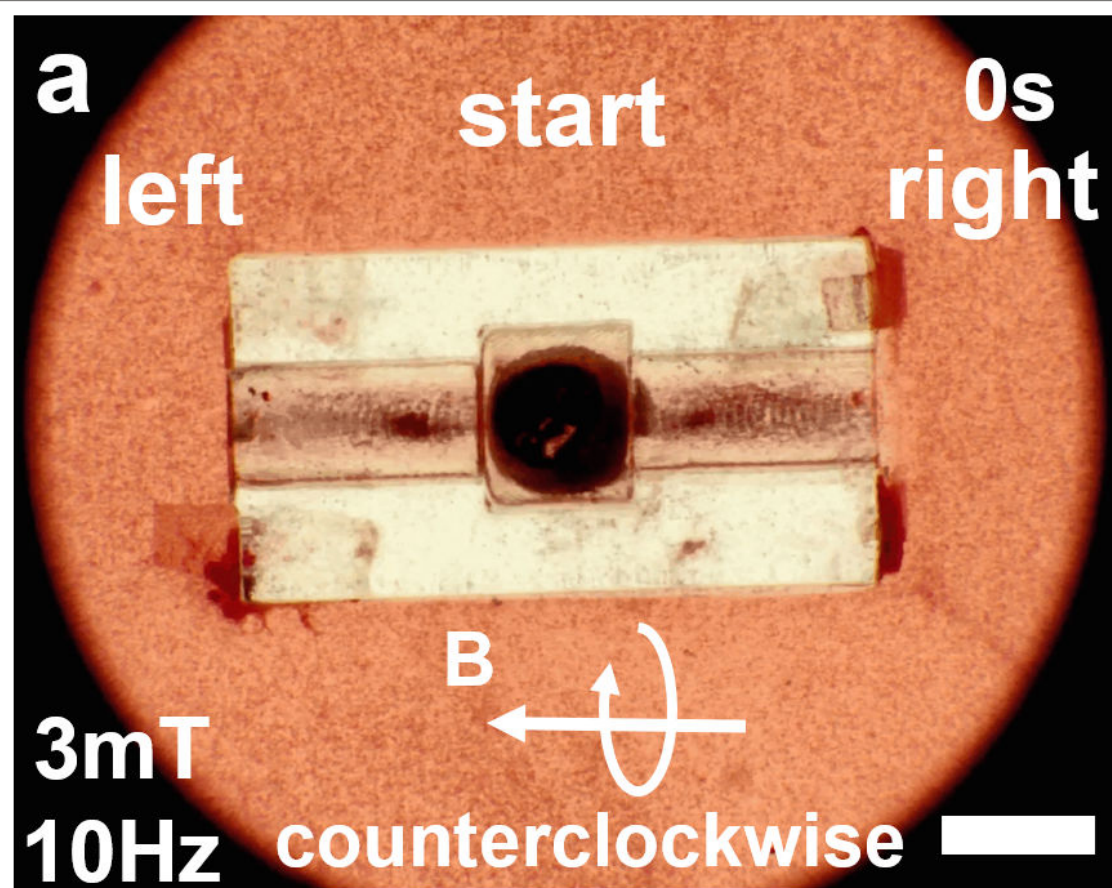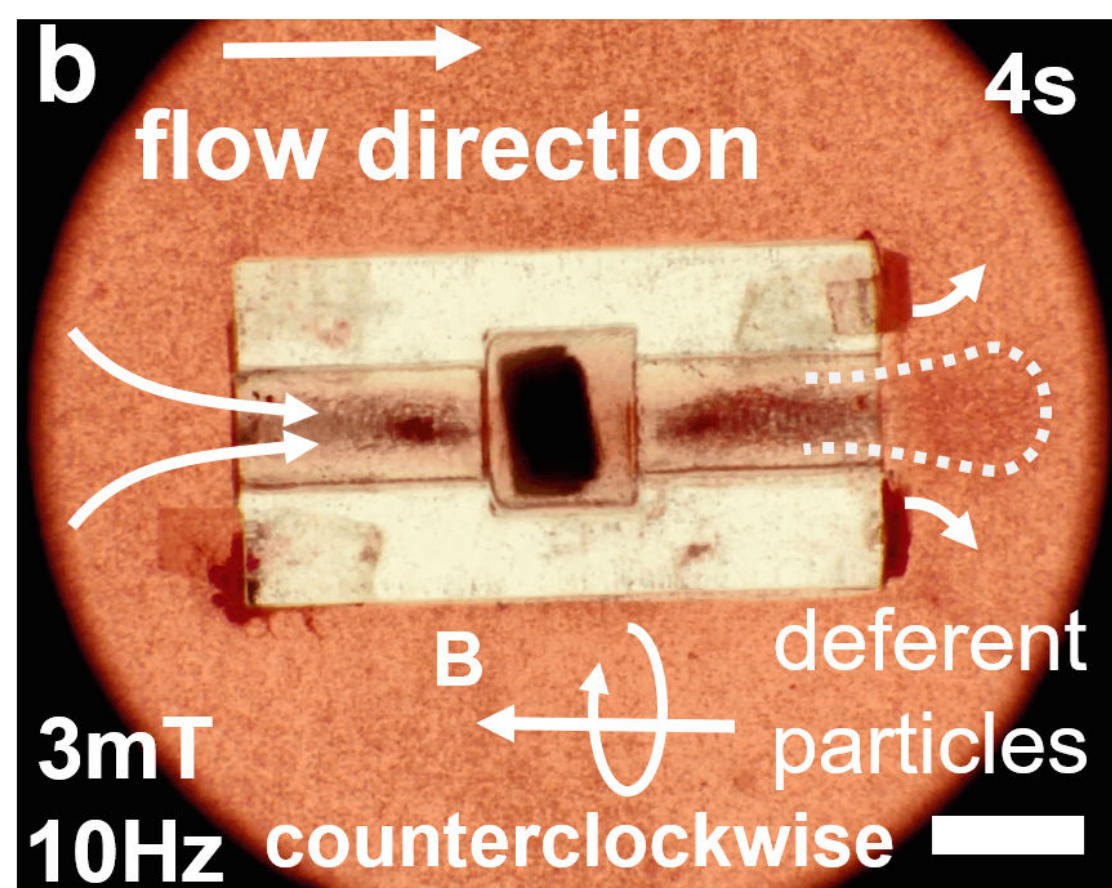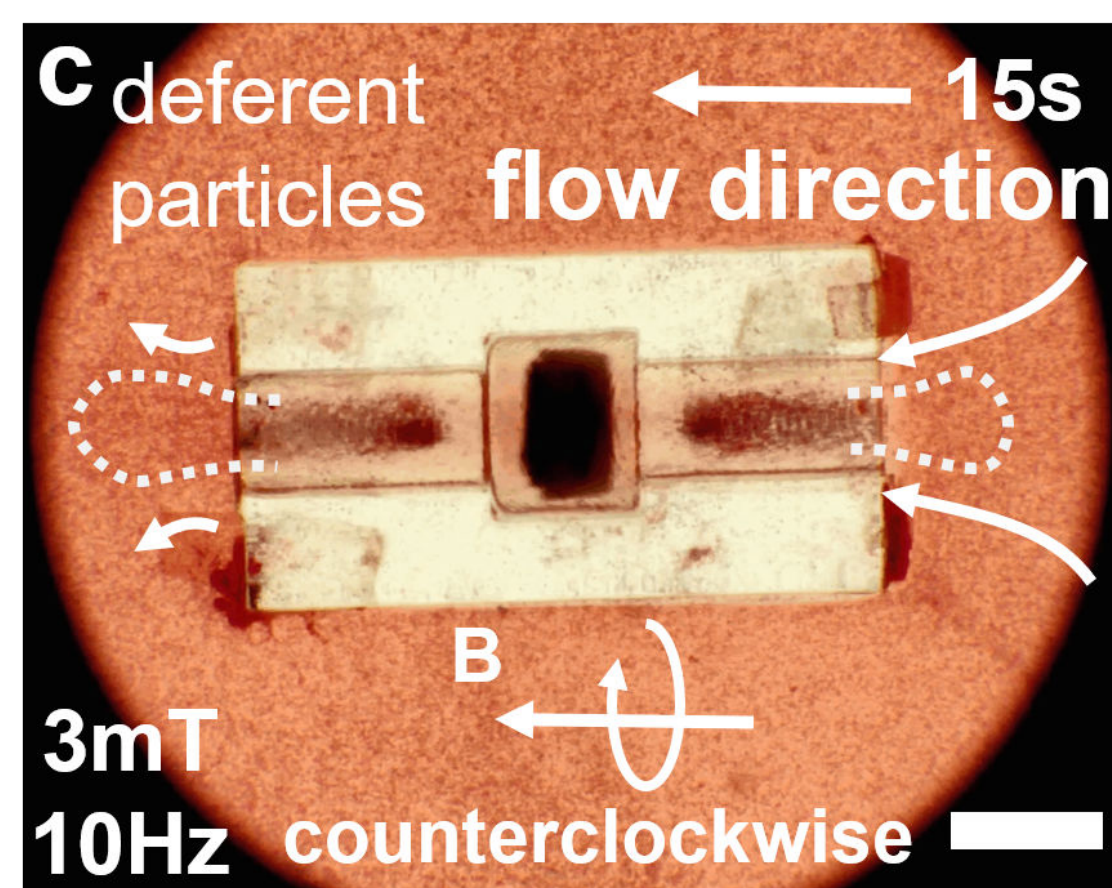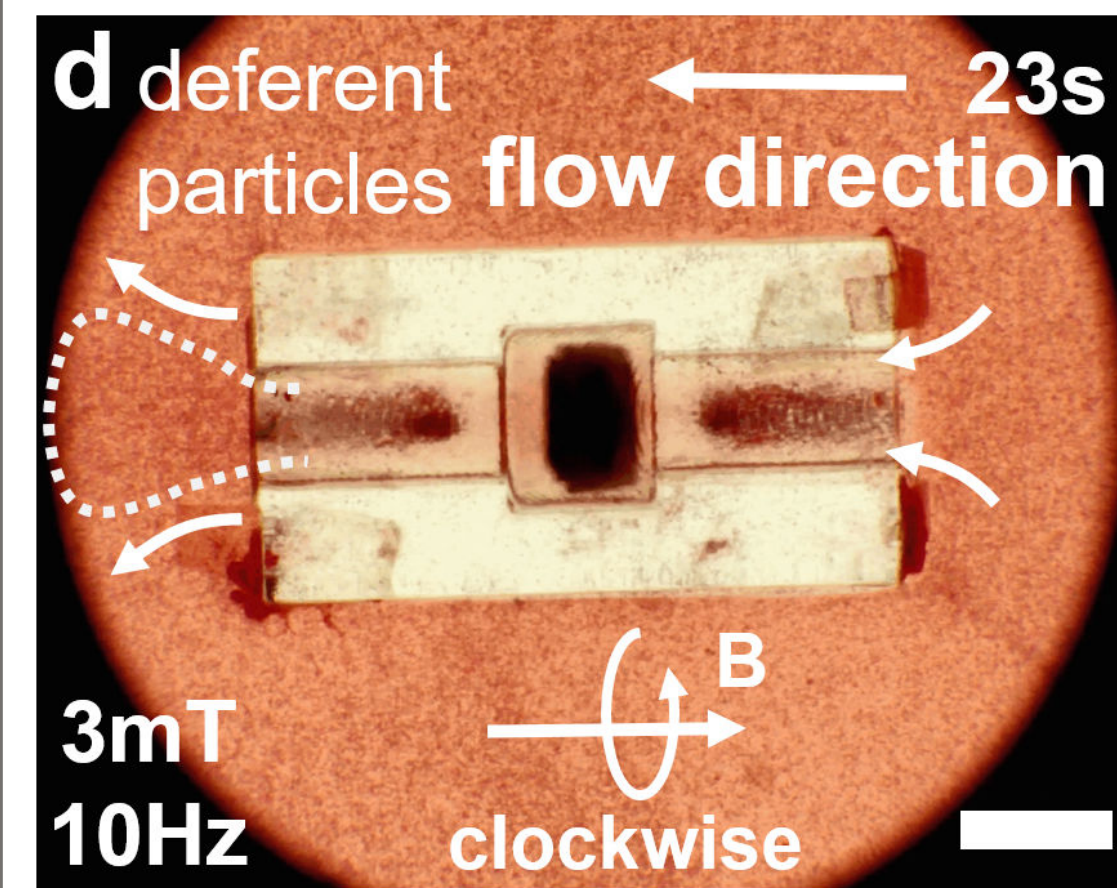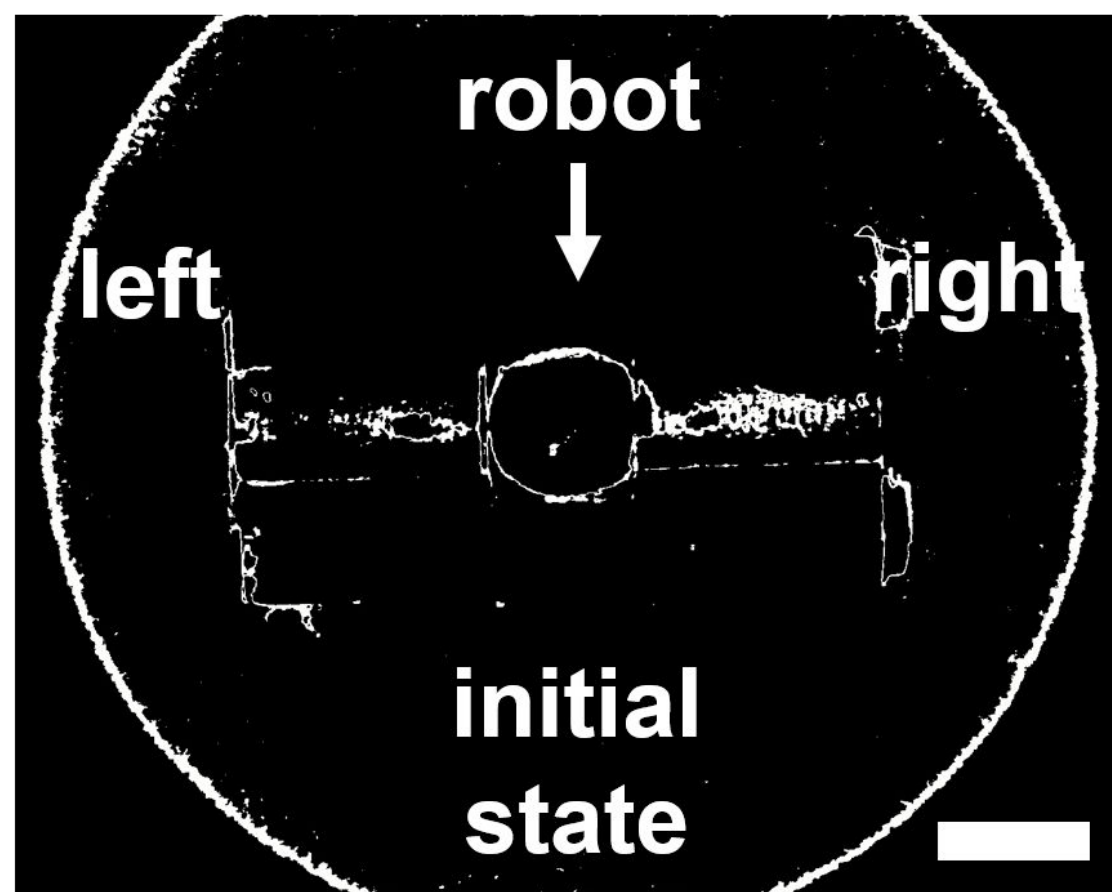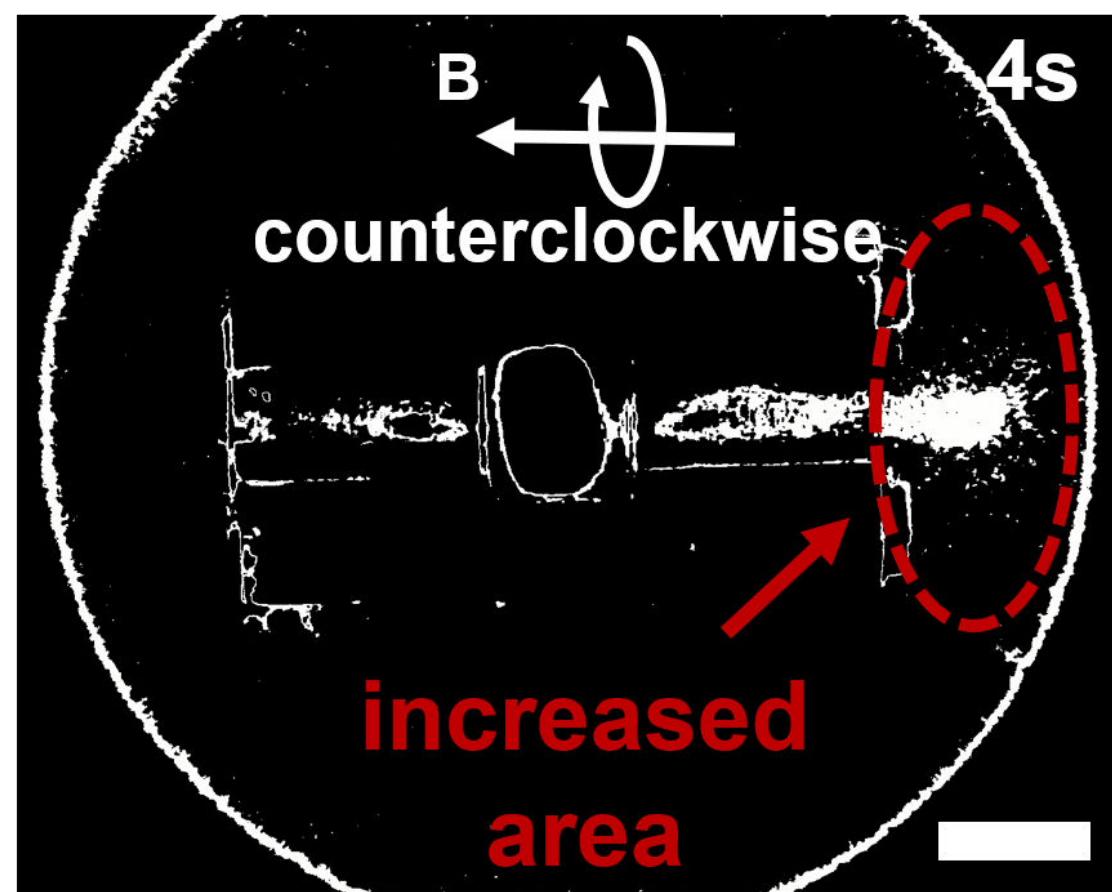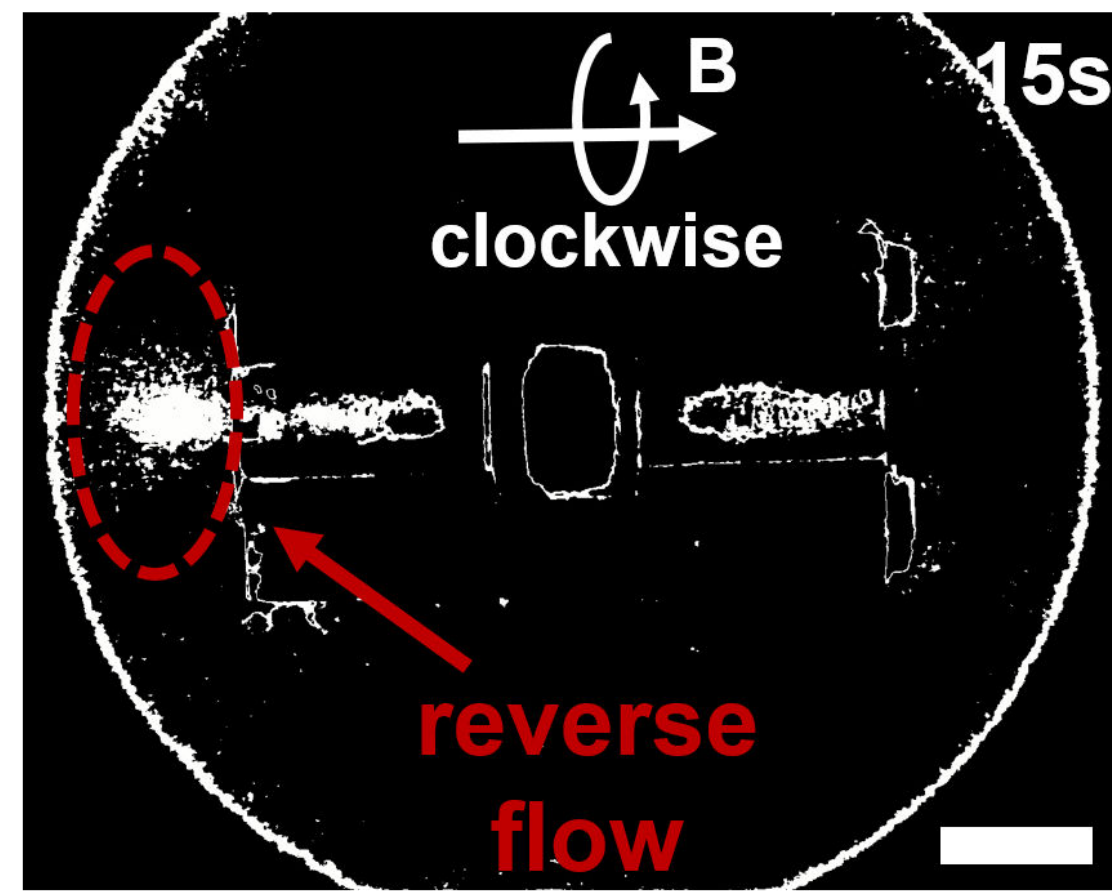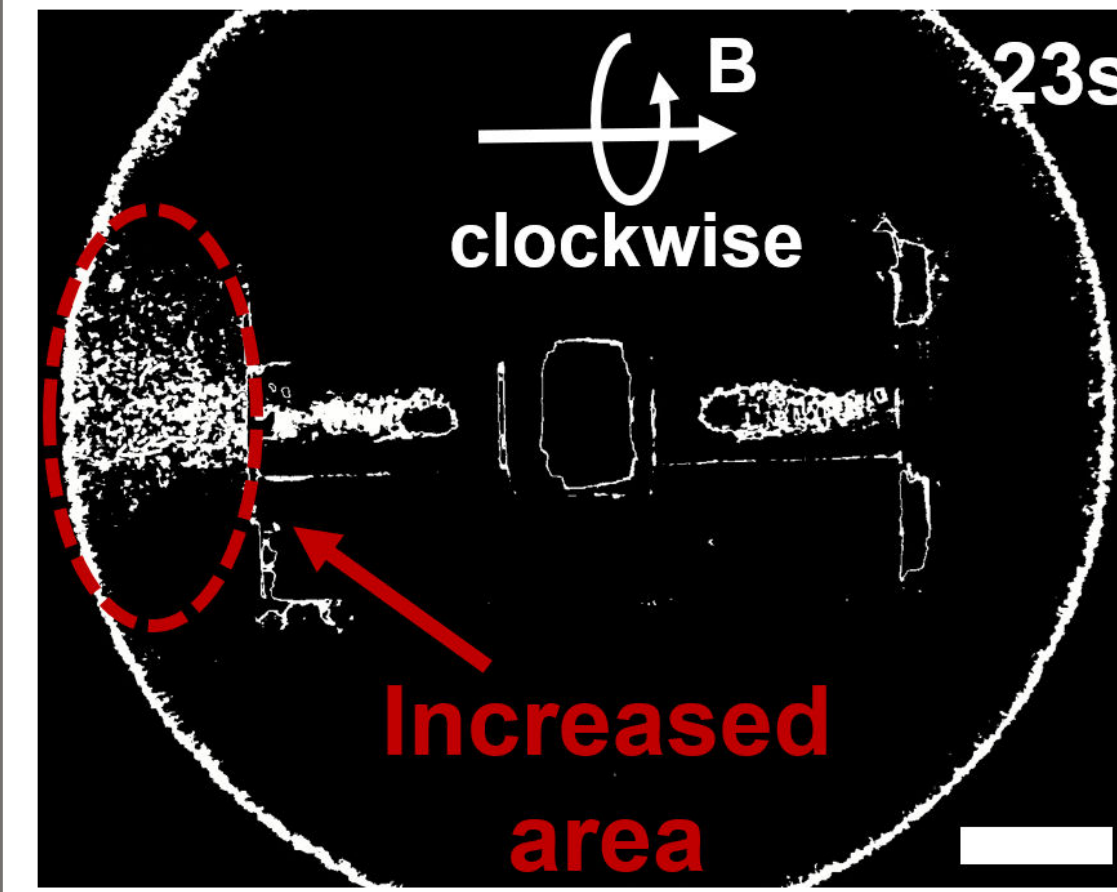

Supplement: Supplementary 1 — Movies S1 to S7 Fig. S1 [file cbsystems.0235.f1.zip › Supplementary Figure S1.pdf]
